# A simple robot suggests trunk rotation is essential for emergence of inside leading limb during quadruped galloping turns

**DOI:** 10.3389/fnbot.2025.1628368

**Published:** 2025-10-23

**Authors:** Tomoe Maeta, Shoei Hattori, Takeshi Kano, Akira Fukuhara, Akio Ishiguro

**Affiliations:** ^1^Research Institute of Electrical Communication, Tohoku University, Sendai, Japan; ^2^Graduate School of Biomedical Engineering, Tohoku University, Sendai, Japan; ^3^Graduate School of Electrical Engineering, Tohoku University, Sendai, Japan; ^4^Division for Interdisciplinary Advanced Research and Education, Tohoku University, Sendai, Japan; ^5^Japan Society for the Promotion Science, Tokyo, Japan; ^6^School of Systems Information Science, Future University Hakodate, Hakodate, Japan

**Keywords:** turning behavior, non-steady locomotion, decentralized control, quadrupedal locomotion, robot experiment, galloping gait

## Abstract

During turning maneuvers in the galloping gait of quadruped animals, a strong relationship exists between the turning direction and the sequence in which the forelimbs make ground contact: the outer forelimb acts as the “trailing limb” while the inner forelimb serves as the “leading limb.” However, the control mechanisms underlying this behavior remain largely unclear. Understanding these mechanisms could deepen biological knowledge and assist in developing more agile robots. To address this issue, we hypothesized that decentralized interlimb coordination mechanism and trunk movement are essential for the emergence of an inside leading limb in a galloping turn. To test the hypothesis, we developed a quasi-quadruped robot with simplified wheeled hind limbs and variable trunk roll and yaw angles. For forelimb coordination, we implemented a simple decentralized control based on local load-dependent sensory feedback, utilizing trunk roll inclination and yaw bending as turning methods. Our experimental results confirmed that in addition to the decentralized control from previous studies which reproduces animal locomotion in a straight line, adjusting the trunk roll angle spontaneously generates a ground contact sequence similar to gallop turning in quadruped animals. Furthermore, roll inclination showed a greater influence than yaw bending on differentiating the leading and trailing limbs. This study suggests that physical interactions serve as a universal mechanism of locomotor control in both forward and turning movements of quadrupedal animals.

## 1 Introduction

Animals navigate unpredictable terrains while frequently changing their direction of movement to evade predators, catch prey, and achieve reproductive success (Daley, 2016; Wilson et al., 2018). Existing research has primarily focused on mechanisms under steady and periodic conditions, such as straight-line movement on level terrain, to comprehend the fundamental mechanisms underlying this skillful locomotion (Shik, 1969; Grillner, 1975). Significant progress has been made in understanding these steady locomotion patterns; in recent years, studies on non-steady locomotion, such as turning and recovery from perturbations, have also gained attention (Daley, 2016; Sponberg et al., 2023). However, from the perspective of control mechanisms, our understanding of non-steady locomotion remains limited. Understanding the control mechanisms of non-steady locomotion is expected to enhance biological knowledge and help develop more agile robots.

**Figure 1:**
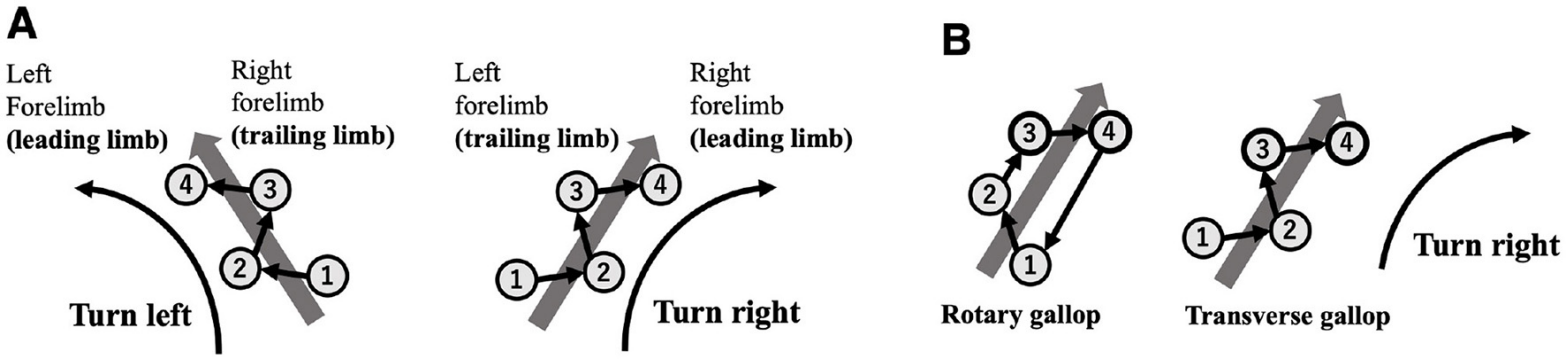
**(A)** Relationship between turning direction and forelimb footfall sequence during transverse gallop. The numbers shown in the figure represent the footfall sequence (1 and 2: hindlimbs; 3 and 4: forelimbs). The first limb to contact the ground is defined as the trailing limb, and the next limb to contact the ground is defined as the leading limb. When turning, the outer limb tends to act as the trailing limb, and the inner limb tends to act as the leading limb. **(B)** Regardless of different gallop type (rotary gallop and transverse gallop), it is consistently observed that the inner forelimb takes on the role of the leading limb during turns.

This study focuses on the high-speed turning motion of quadrupeds–an intriguing example of non-steady movement. Notably, when quadrupeds are running at their highest speeds, they tend to use a stride pattern known as an asymmetrical, four-beat gait called a gallop, where turning direction is strongly correlated with the order of forelimb contact. During a galloping turn, the outer forelimb lands first as the “trailing limb” while the inner forelimb lands later and initiates the leap as the “leading limb” (e.g., in a rightward turn, the left forelimb serves as the trailing limb and the right forelimb acts as the leading limb; Hildebrand, 1977; Figure 1A). Furthermore, when switching turning directions, quadrupeds exhibit a behavioral pattern known as a “lead change” in which the order of limb contact reverses (Back and Clayton, 2013). Gallop can be classified into rotary gallop and transverse gallop, depending on the sequence of footfalls (Biancardi and Minetti, 2012), and different species and speeds determine which type is used. Regardless of the gallop type, it is consistently observed that the inner forelimb takes on the role of the leading limb during turns (Parkin et al., 2006; Williams and Norris, 2007; Ichikawa et al., 2018; Higurashi et al., 2024; Figure 1B).

The behaviors described above have been examined from a biomechanical perspective in several studies. For instance, as part of research on the dynamical characteristics of animals, one study investigated the ground reaction forces in both the vertical and lateral directions of the inner and outer forelimbs of a horse running in a circular trajectory at a canter (also known as a three-beat gallop, which is an asymmetrical gait; Camus et al., 2012). Another study examined galloping horses on an elliptical track, comparing the duty factors of the inner and outer limbs during curved segments (Parkes et al., 2020). (Hildebrand 1987) suggested that using the inner forelimb as the leading limb during turns allows the animal to maintain support while moving closer to the running trajectory. However, there is still little known about the control mechanism underlying these behaviors.

This research aims to elucidate the control mechanisms underlying adaptive behaviors in galloping turns. We hypothesized that a decentralized control mechanism and trunk movement to generate turning motion are essential components for the emergence of the forelimb footfall sequence observed in gallop turning. This is because decentralized control mechanisms–such as central pattern generators (CPGs) and local sensory feedback–are suggested to be essential for generating locomotor patterns in response to change their situations and environments (Iida and Ijspeert, 2016; Ijspeert, 2017). Since the trunk roll inclination and yaw bending are observed in actual animal turning (Eilam, 1994; Egenvall et al., 2023), so we hypothesized footfall sequence in gallop tuning are naturally generated by compound these decentralized control and trunk motion. To adress this issue, we adopt a decentralized control mechanism, previously proposed by our research group (Owaki et al., 2013; Owaki and Ishiguro, 2017). This is a simple and abstract model based on CPG and local sensory feedback, and it can effectively generate speed-dependent gait transitions through physical interactions and descending modulation. We built a robotic platform and implemented a decentralized inter-limb coordination mechanism from a previous study (Owaki et al., 2013), which enabled locomotion in a straight line. We then extended this mechanism to include trunk movements for body flexion and evaluated the resulting effects. Consequently, we report that an inside leading limb in galloping gaits during turning was spontaneously organized in accordance with trunk roll rotation.

The remainder of this paper is structured as follows. Section 2 describes the robotic platform, along with the autonomous distributed control using local feedback and trunk control. Section 3 presents the experimental setup and results. Section 4 discusses the findings and suggests suggestions for future research.

## 2 Robot

This study investigates the control mechanisms of quadrupeds during galloping turns using a synthetic approach based on mathematical modeling (Gravish and Lauder, 2018). This section describes the mechanical model of a legged robot, which incorporates roll and yaw degrees of freedom in its trunk, and proposes a decentralized control scheme inspired by animal locomotion.

### 2.1 Mechanical system

**Figure 2:**
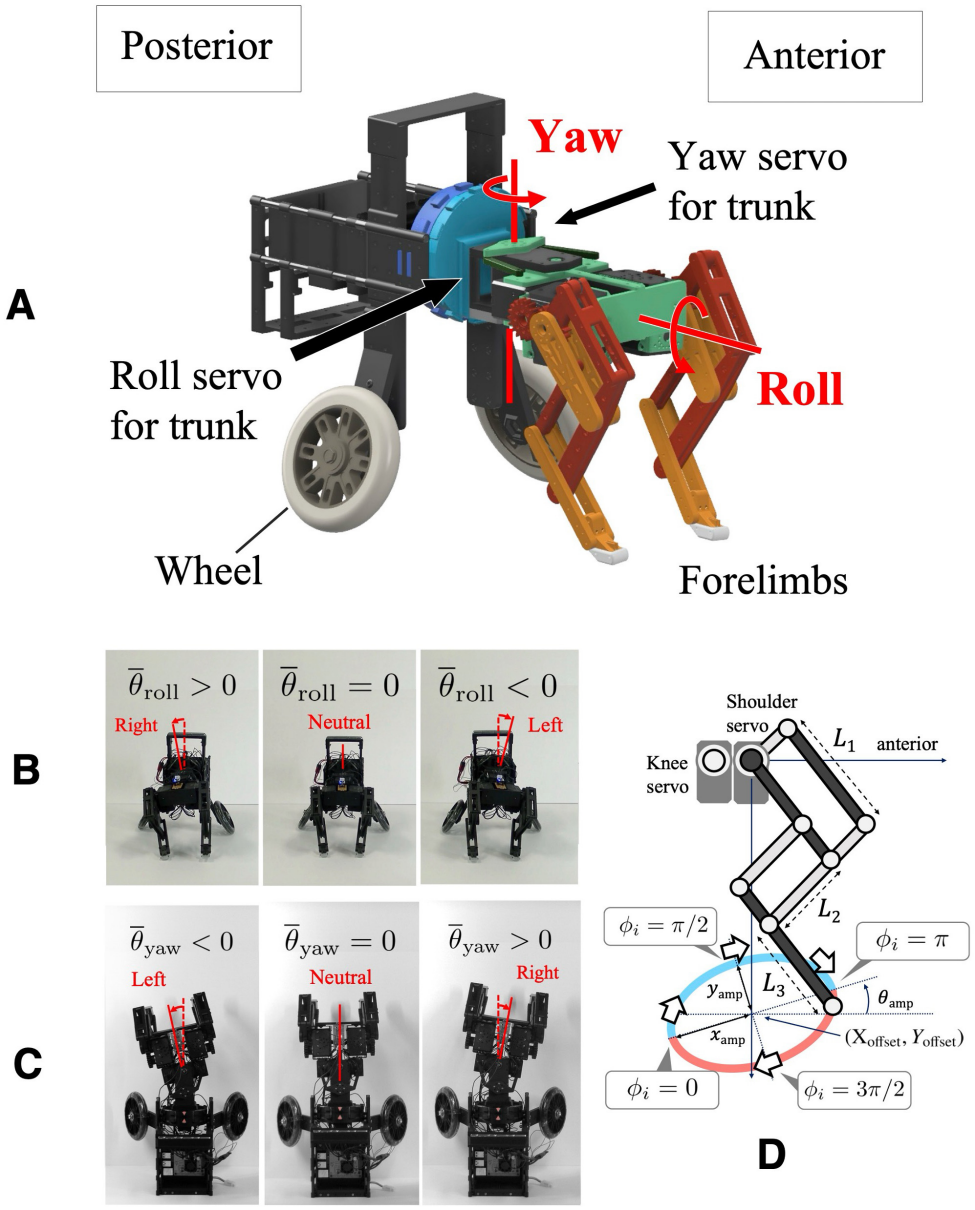
**(A)** Overview of the developed robot. The structure consists of a front module with a pair of legs and a hind module with a pair of wheels. **(B)** Robot front view showing the trunk roll change mechanism, allowing for lateral tilting. **(C)** Robot top view showing the trunk yaw change mechanism, allowing for lateral bending. **(D)** Schematics of the pantograph limb structure.

To focus on forelimb coordination, we developed a quasi-quadruped robot in which the hindlimbs are replaced with wheels. This design is based on the assumption that the hindlimb sequence has a lower impact on gallop turning. This simplification is based on biological findings indicating that the forelimb sequence remains consistent while the hindlimb footfall sequence varies across different gallop gaits during turning (Parkin et al., 2006; Ichikawa et al., 2018) (Figure 1B). The robot platform used in this study has the following specifications: 200 [mm] distance between forelimbs and wheels, 120 [mm] width, 180 [mm] leg length in standing posture, and an approximate mass of 2.5 [kg]. The structure consists of a front module with a pair of legs and a hind module with a pair of wheels (Figure 2A). The hind module includes a counterweight to facilitate forelimb jumping. The trunk has roll and yaw degrees of freedom, each actuated by a servo motor (ROBOTIS: DYNAMIXEL XM430-W350R; Figures 2B, C), allowing stable locomotion with fixed roll and yaw angles.

Each leg is equipped with three servo motors (ROBOTIS: DYNAMIXEL XM430-W350R) that provide flexion-extension, fore-aft swinging motions (Figure 2D), and abduction-adduction. The motor responsible for abduction-adduction is fixed in the parasagittal plane to focus on trunk motion, and each leg is tilted inward by 5 [deg] to enable the robot to run straight. The pantograph leg mechanism is based on a previous study (Fukuhara et al., 2020b), with *L1, L2*, and *L3* representing the proximal, intermediate, and distal segment lengths, respectively. In this study, the segment lengths used in this study are *L1*: 75 [mm], *L2*: 68 [mm], and *L3*: 50 [mm]. A single-board computer (Raspberry Pi Foundation: Raspberry Pi 4 Model B) handles motor control and sensor acquisition. Power is supplied by an 11.1 [V] LiPo battery, enabling untethered operation.

### 2.2 Control system

In our previous work, we proposed a decentralized control framework based on local sensory feedback that allows for gait transitions dependent on locomotion speed and gait selection based on body properties. This model incorporates local feedback mechanisms based on ground reaction forces (GRF) at each leg and is realized through decoupled CPGs. Assuming that each leg is equipped with a phase oscillator ϕ_*i*_, the foot position is controlled along a predefined trajectory in the sagittal plane according to the oscillator's phase (Figure 2D). Depending on the phase ϕ_*i*_, the leg is in the swing phase when 0 < ϕ_*i*_ ≤ π, and in the stance phase when π < ϕ_*i*_ ≤ 2π. The target foot positions ${\bar{x}}_{i}$ and ${\bar{y}}_{i}$ are given by the following equations:


(1) $$\begin{array}{l} \{\begin{array}{c} {\bar{x}}_{i}=X_{\text{offset}}+x_{\text{amp}}\cos\phi_{i}\\ {\bar{y}}_{i}=Y_{\text{offset}}+y_{\text{amp}}\sin\phi_{i}. \end{array} \end{array}$$

The parameters *X*_offset_ and *Y*_offset_ represent the center of the foot trajectory, while *x*_amp_ and *y*_amp_ are the amplitudes in the fore-aft and vertical directions, respectively. Furthermore, this foot trajectory is tilted upward by θ_amp_ (Figure 2D). The temporal evolution of the oscillator phase ϕ_*i*_ [rad] implemented in each leg is described by the following equation (Owaki et al., 2013):


(2) $$\begin{array}{l} \overset{∙}{\phi_{i}}=\omega -\sigma N_{i}\cos\phi_{i}\;\left(i=\text{left},\text{right}\right) \end{array}$$

The subscript *i* denotes the leg index. In the following equation, the first term ω [rad/s] represents the intrinsic angular frequency of the oscillator. The second term accounts for local sensory feedback, where σ is a positive constant representing the feedback gain, and *N*_*i*_ denotes the ground reaction force. Physically, this second term ensures that the oscillator phase is held at 3π/2 when a leg is in contact with the ground (*N*_*i*_ > 0), maintaining the leg in the stance phase. Using this simple interlimb coordination model, various gaits were reproduced either by changing the parameter ω–which governs locomotion speed–or by leveraging changes in the physical properties of the robot (Owaki et al., 2013).

The motors used in the robot can provide current values, which are proportional to the motor torque. Therefore, in the present experiment, a filtered value of the shoulder joint motor current was used as a virtual GRF *N*_*i*_. The method for acquiring the GRF is described in the Supplementary material.

Control inputs were applied to modulate the roll and yaw angles of the trunk, thereby enabling turning maneuvers. In particular, the variable ${\bar{\theta }}_{\text{roll}}$ was used as the target angle for the servo motor responsible for trunk roll. When ${\bar{\theta }}_{\text{roll}}=0$, the body is in a neutral position; ${\bar{\theta }}_{\text{roll}}>0$ tilts the body to the right, and ${\bar{\theta }}_{\text{roll}}<0$ tilts it to the left (Figure 2B). Likewise, the variable ${\bar{\theta }}_{\text{yaw}}$ was used as the target angle for the servo motor responsible for trunk yaw bending. When ${\bar{\theta }}_{\text{yaw}}=0$, the body is in a neutral position; ${\bar{\theta }}_{\text{yaw}}>0$ causes a rightward bend, and ${\bar{\theta }}_{\text{yaw}}<0$ results in a leftward bend (Figure 2C). The values of ${\bar{\theta }}_{\text{roll}}$ and ${\bar{\theta }}_{\text{yaw}}$ were adjusted depending on the experimental conditions.

## 3 Experiments

During turning maneuvers, quadrupedal animals are known to exhibit both trunk yaw bending and roll tilting (Egenvall et al., 2023). This study aims to evaluate the relative contribution of each component–trunk roll tilt and yaw bending–to the emergence of characteristic footfall sequence patterns observed during galloping turns by systematically combining them with the proposed model.

### 3.1 Experimental setup

In this study, experiments were conducted under the following conditions. The robot was operated using the control law described in Section 2.2. After 6 [s] of operation, locomotion was initiated under a combination of predefined values for ${\bar{\theta }}_{\text{roll}}$ and ${\bar{\theta }}_{\text{yaw}}$. Following the change in trunk orientation, the robot continued to operate until 60 [s].

The value of ${\bar{\theta }}_{\text{roll}}$ ranged from -10 [deg] to 10 [deg], and that of ${\bar{\theta }}_{\text{yaw}}$ ranged from -10 [deg] to 10 [deg]. The range was determined by referring to the mean angle for thoracolumbar of quadrupeds during circling (Egenvall et al., 2023). A total of 25 experimental conditions (5 × 5 combinations) were tested, and three trials were conducted for each condition. The initial condition was defined as ${\bar{\theta }}_{\text{roll}}=0\;\left[\text{deg}\right]$ and ${\bar{\theta }}_{\text{yaw}}=0\;\left[\text{deg}\right]$. For the initial setup, we positioned the robot facing inward so that it would be within the camera's field of top view. The robot initial parameters were not changed. The robot started from a stationary position on the ground.

**Figure 3:**
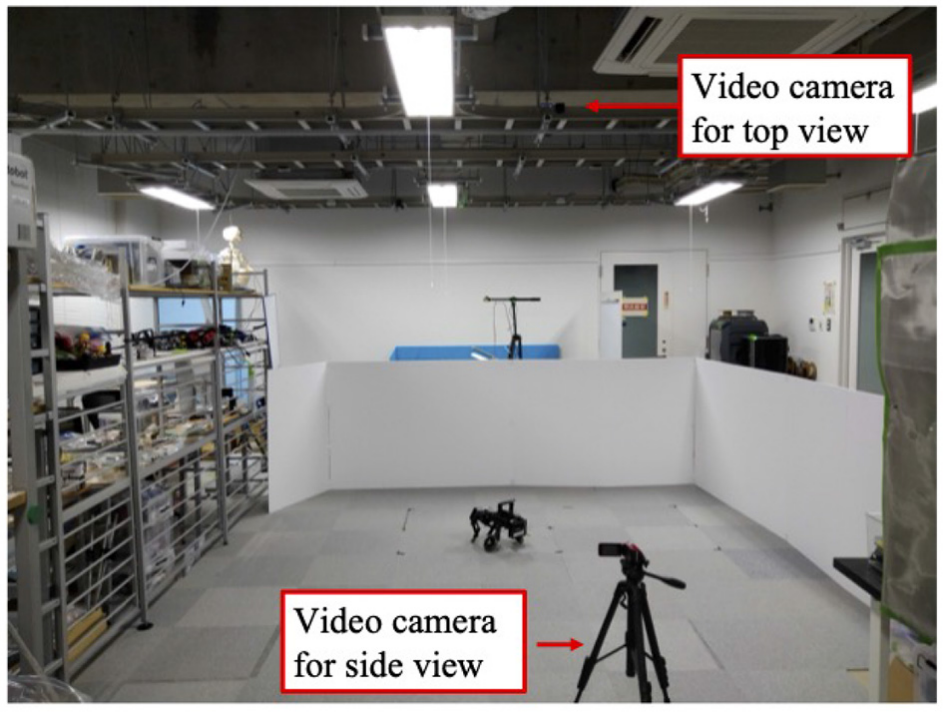
The experimental environment.

The parameters used in the robot experiments are listed in Table 1 and the experimental setup is illustrated in Figure 3. Experiments were conducted on a carpeted surface, and the robot's motion was recorded using video cameras mounted on the ceiling and from the lateral side.

**Table 1:** Parameters for the robot experiments.

**Parameters**	**Values**	**Units**
ω	12.0	[rad/s]
σ	0.09	[rad/(mA·s)]
*X*_offset_	0.00	[m]
*Y*_offset_	0.16	[m]
*x*_amp_	0.06	[m]
*y*_amp_	0.03	[m]
θ_amp_	0.20	[rad]

### 3.2 Performance measure

In this experiment, we evaluated the performance of the robot based on the phase difference between the forelimbs and the movement trajectory and its curvature. The phase difference Δϕ was defined as the difference between the phase of the left forelimb (LF) and that of the right forelimb (RF). The criteria for evaluating Δϕ were set as follows:

1. Δϕ = ϕ_LF_ − ϕ_RF_ = 1.0π [rad]: anti-phase condition

2. Δϕ = ϕ_LF_ − ϕ_RF_ < 1.0π [rad]: right forelimb is the leading limb

3. Δϕ = ϕ_LF_ − ϕ_RF_ > 1.0π [rad]: left forelimb is the leading limb.

We define the range of Δϕ as [0, 2π] [rad]. As Δϕ approaches 1.0π [rad], the interlimb coordination tends toward anti-phase, while values close to 0 or 2.0π [rad] indicate in-phase coordination. We calculated the average phase difference $\bar{\Delta \phi }$ to evaluate the phase relationship between the forelimbs. This was obtained by averaging the values of Δϕ measured at each time point within the 50–60 [s] interval, during which the robot's gait was considered to have converged.

The curvature of the robot's trajectory was determined by calculating the turning radius and then taking its reciprocal. Turning direction was identified from overhead video footage, where positive values represent right turns and negative values represent left turns. An overhead camera tracked the position of a marker on the robot's torso using “Tracker,” a free video analysis and modeling tool (Open Source Physics, 4 27), to calculate the turning radius. The method for calculating the turning radius is described in the Supplementary material.

The average of the three trials was used for the phase difference of the forelimbs and the curvature.

### 3.3 Result

**Figure 4:**
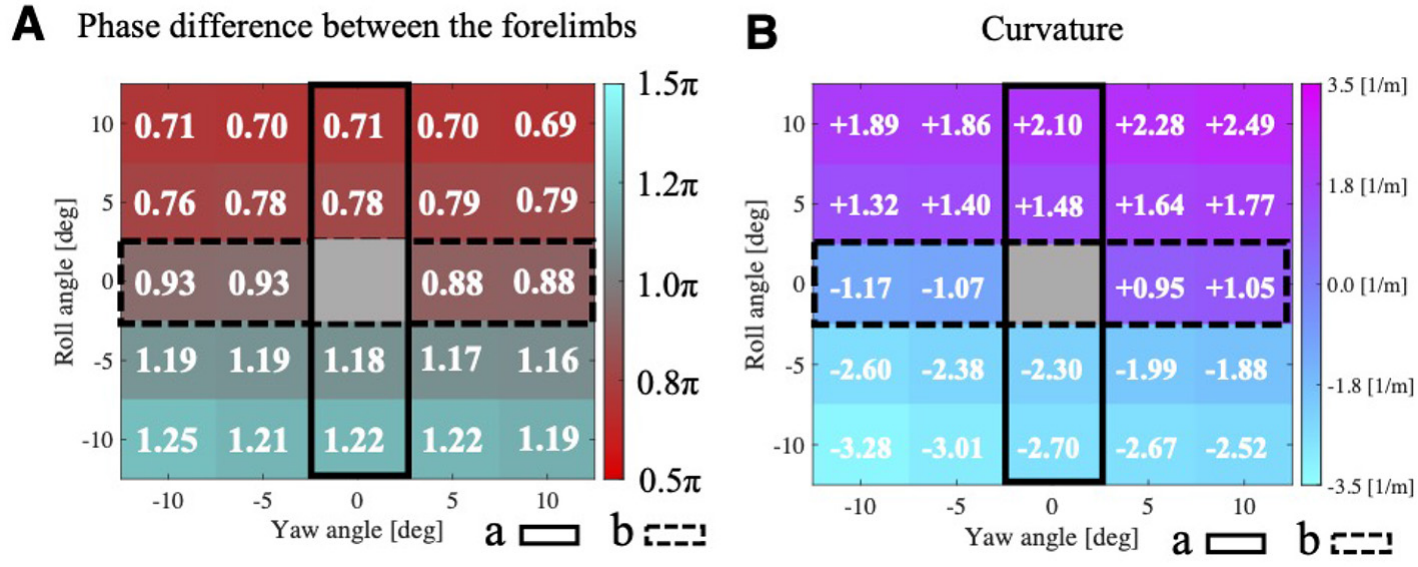
Colormaps of **(A)** forelimb phase differences and **(B)** robot path curvature (positive = right turn, negative = left turn) resulting from simultaneous 5 [deg] changes in trunk roll and yaw angle. Trunk roll and yaw angles were varied from –10 [deg] to +10 [deg] in 5 [deg] increments. The case where both roll angle and yaw angle were 0 [deg] was excluded because we were unable to prepare a sufficiently long straight course; this region is highlighted in gray for clarity.

We evaluated the phase difference of the robot's forelimbs (Figure 4A) and curvature (Figure 4B) while varying the roll and yaw of the trunk in 5[deg] increments from −10 [deg] to 10 [deg] each. Positive curvature values indicate a right turn, while negative values indicate a left turn. The case where both roll angle and yaw angle were 0 [deg] was excluded because we were unable to prepare a sufficiently long straight course; this region is highlighted in gray for clarity.

The tilting of the trunk's roll angle had a more significant impact on a smaller phase difference than the bending of the yaw angle (Figure 4A). In Region (a) of Figure 4A, where the trunk's yaw angle was 0[deg] and only the trunk's roll angle varied, the phase difference between the forelimbs tended to decrease as the trunk's roll angle increased. Conversely, in Region (b) of Figure 4A, where the trunk's roll angle was 0 [deg] and only the trunk's yaw angle varied, an increase in the trunk's yaw angle did not lead to a smaller forelimbs' phase difference, unlike the case where the trunk was tilted in the roll direction. Notably, these trends were observed on both the left and right sides.

Regarding the turning direction, the robot tended to turn toward the side to which the trunk's roll was inclined. Figure 4B illustrates the curvature when the parameters of roll inclination and yaw bending were varied at regular intervals. The robot turned toward the side of the roll inclination when the roll inclination and yaw bending were in opposite directions (e.g., roll tilted right, and yaw bent left; Supplementary Video S1). Furthermore, even for the same angle of roll inclination, the curvature tended to be larger when the side of the roll inclination and the side of the yaw bending were the same (e.g., roll tilted right, and yaw bent right).

In this 5 × 5 condition space, we define Condition 1 as $\left({\bar{\theta }}_{\text{roll}},{\bar{\theta }}_{\text{yaw}}\right)=\left(-10,0\right)$ and Condition 2 as $\left({\bar{\theta }}_{\text{roll}},{\bar{\theta }}_{\text{yaw}}\right)=\left(0,-10\right)$. This setup is intended to compare the influence of changes in roll angle alone (Condition 1) and changes in yaw angle alone (Condition 2) on the transition of interlimb coordination.

**Figure 5:**
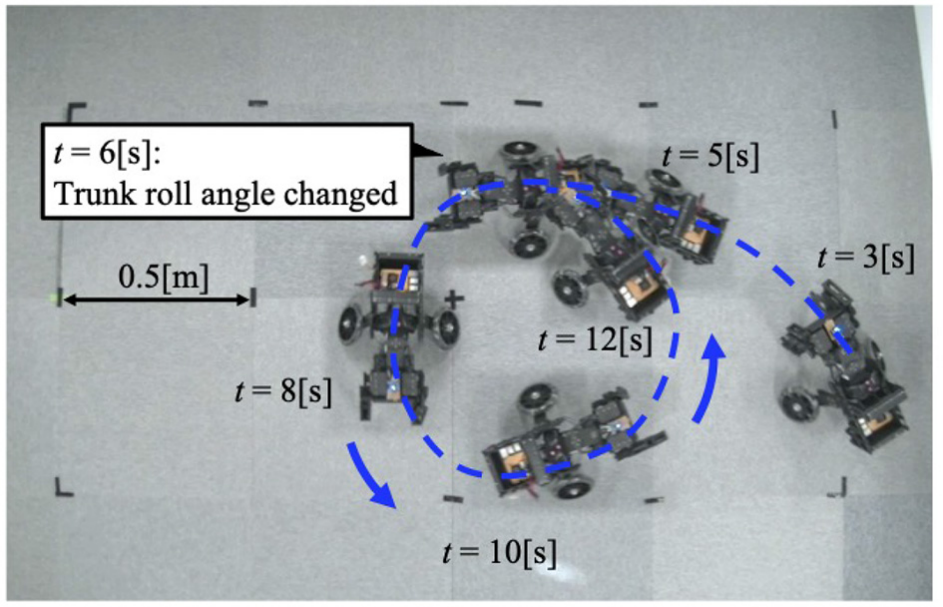
The trajectory of the robot when the trunk roll angle is changed. The robot turned in the direction of the trunk's yaw flexion.

**Figure 6:**
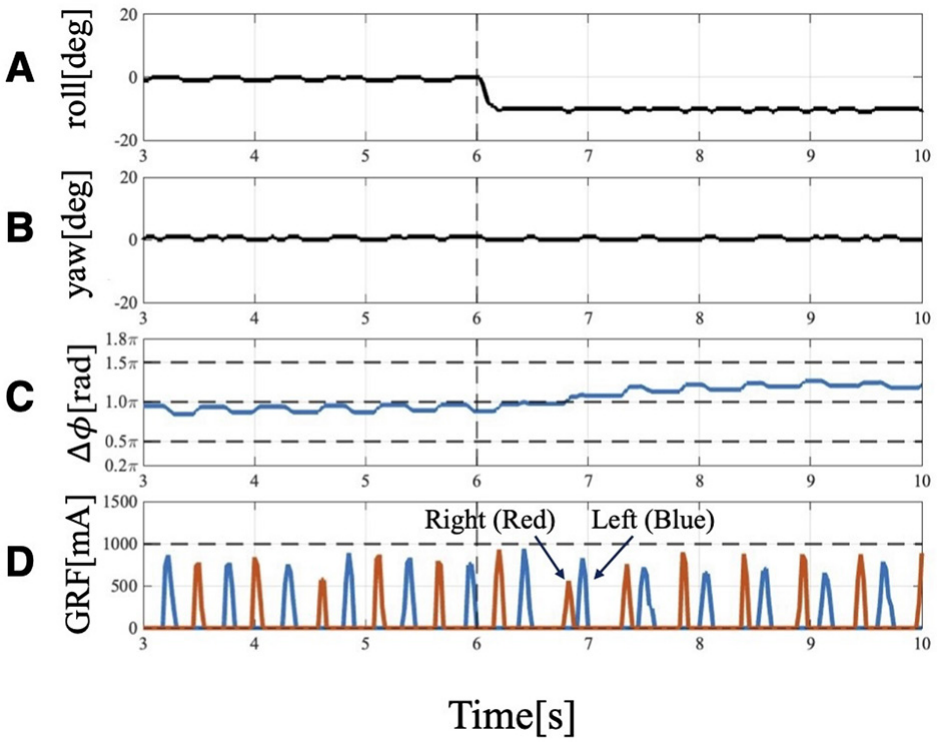
Experimental result of Condition 1: Trunk roll angle changed (${\bar{\theta }}_{\text{roll}}=-10\;[\text{deg}]$), while trunk yaw angle unchanged (${\bar{\theta }}_{\text{yaw}}=0[\text{deg}]$). **(A)** Actual angle of the trunk roll servo motor. **(B)** Actual angle of the trunk yaw servo motor. **(C)** Phase difference of forelimbs. Before 6[s], the phase relation of the forelimbs is 1.0π and anti-phase, but it changes to 1.2π at 4 strides. **(D)** GRF of the forelimbs. Red indicates the right leg, and blue indicates the left leg. When the trunk is tilted, the timing of ground contact is asymmetrical: the right leg (the leg belonging to the outside), which contacts the floor first, becomes the trailing leg, followed by the left leg (the leg belonging to the inside), which becomes the leading leg.

When the robot was operated under Condition 1, it turned to the left (Figure 5). Approximately 6 [s] after the change in trunk roll angle, the following parameters were analyzed: the actual roll angles of the servo motors (Figure 6A), the actual yaw angles of the servo motors (Figure 6B), the phase difference between the forelimbs (Figure 6C), and the ground reaction forces (Figure 6D).

**Figure 7:**
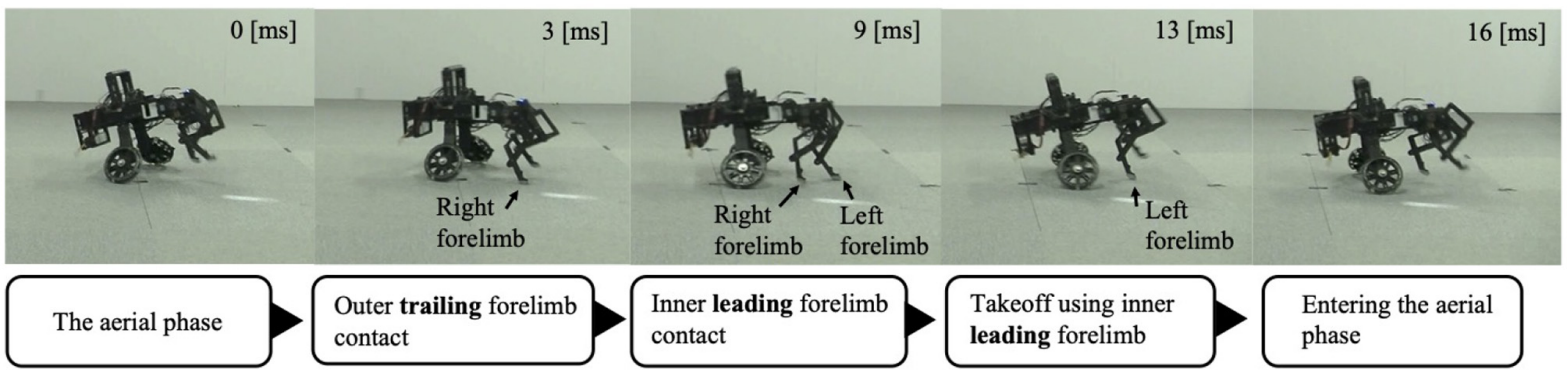
Snapshot of the robot from a lateral view during Condition 1. The right forelimb–the outer trailing limb–contacts the ground first. The left forelimb–the inner leading limb–then contacts the ground before the right forelimb leaves the ground. The robot then pushes off with the left forelimb to enter the aerial phase.

After the trunk roll angle was changed, the forelimb phase relationship in Figure 6C shifted away from 1.0π [rad] and converged after four strides. The footfall sequence depicted in Figure 7D indicates that, following the change in trunk roll angle, the outer leg (red) contacted the ground first, followed by the inner leg (blue), resulting in an asymmetric gait. Figure 7 presents a side-view snapshot of the robot. After the swing phase of both legs, the outer right leg contacted the ground first, and the left forelimb contacted the ground before the right leg had completely lifted off, entering the aerial phase (Supplementary Video S2). Although the footfall sequence was not predetermined, simply changing the trunk's roll angle caused the right forelimb, which is on the outer side of the turning direction, to spontaneously become the trailing limb, while the left forelimb on the inner side became the leading limb.

**Figure 8:**
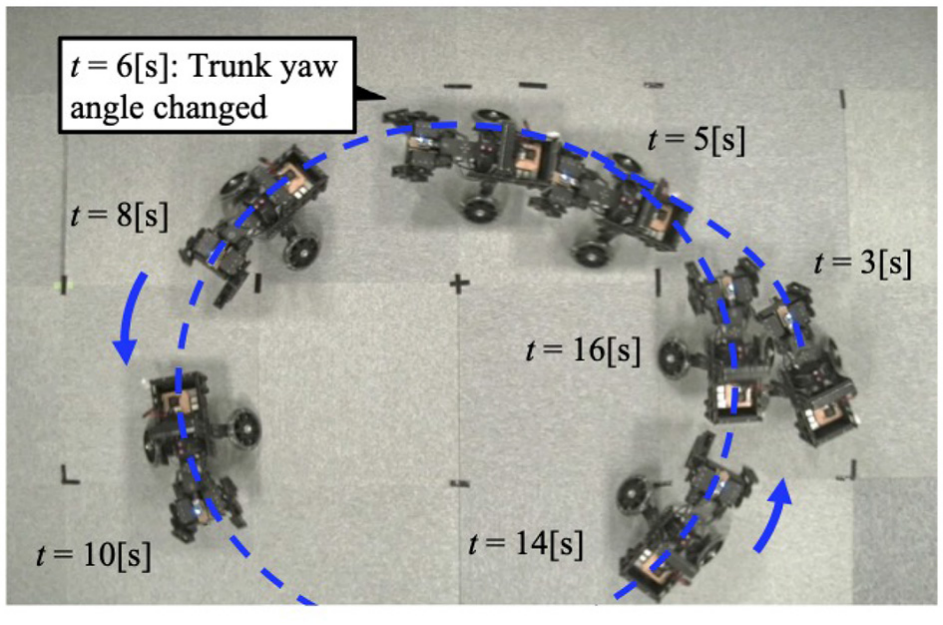
The trajectory of the robot when the trunk yaw angle is changed. The robot turns to the left of the bent yaw and follows a circular path.

**Figure 9:**
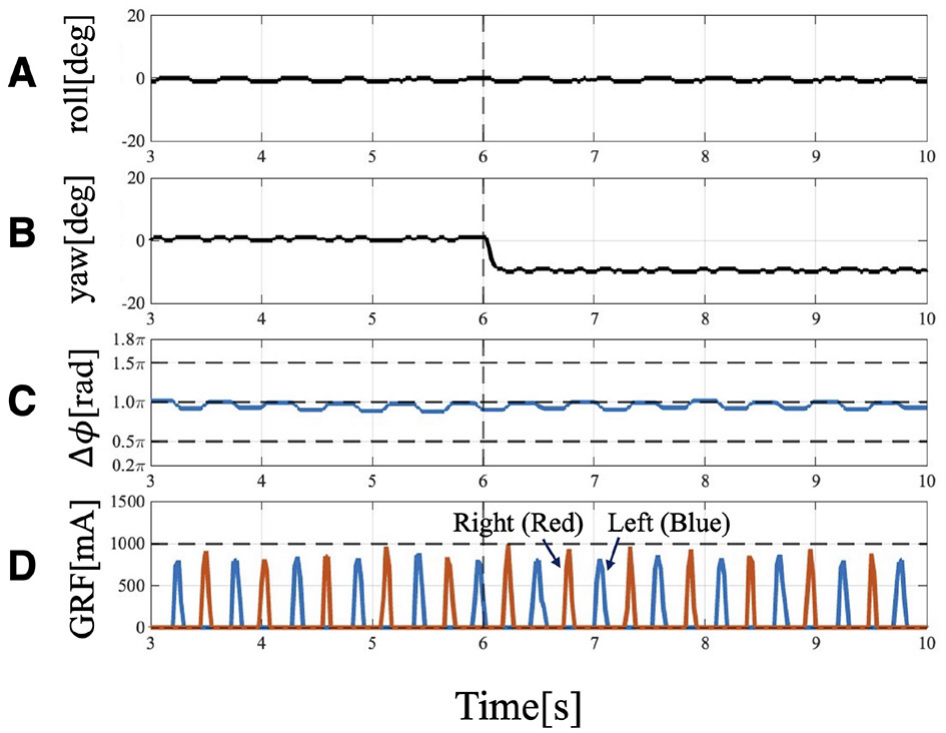
Experimental result of Condition 2: Trunk roll angle unchanged (${\bar{\theta }}_{\text{yaw}}=0[\text{deg}]$), while trunk yaw angle changed (${\bar{\theta }}_{\text{yaw}}=-10\;[\text{deg}]$). **(A)** Actual angle of the trunk roll servo motor. **(B)** Actual angle of the trunk yaw servo motor. **(C)** Phase difference relationship of the forelimbs is 1.0π and they are in anti-phase. Even after the yaw angle is changed, it remains at approximately 1.0π. **(D)** GRF of the forelimbs. Red indicates the right leg, and blue indicates the left leg. Even when the trunk is bent, the timing of ground contact remains symmetrical.

When the robot was operated under Condition 2, it also turned to the left (Figure 8). Approximately 6 [s] after the change in trunk yaw angle, the following parameters were analyzed: the actual roll angles of the servo motors (Figure 9A), the actual yaw angles of the servo motors (Figure 9B), the phase difference between the forelimbs (Figure 9C), and the ground reaction forces (Figure 9D).

**Figure 10:**
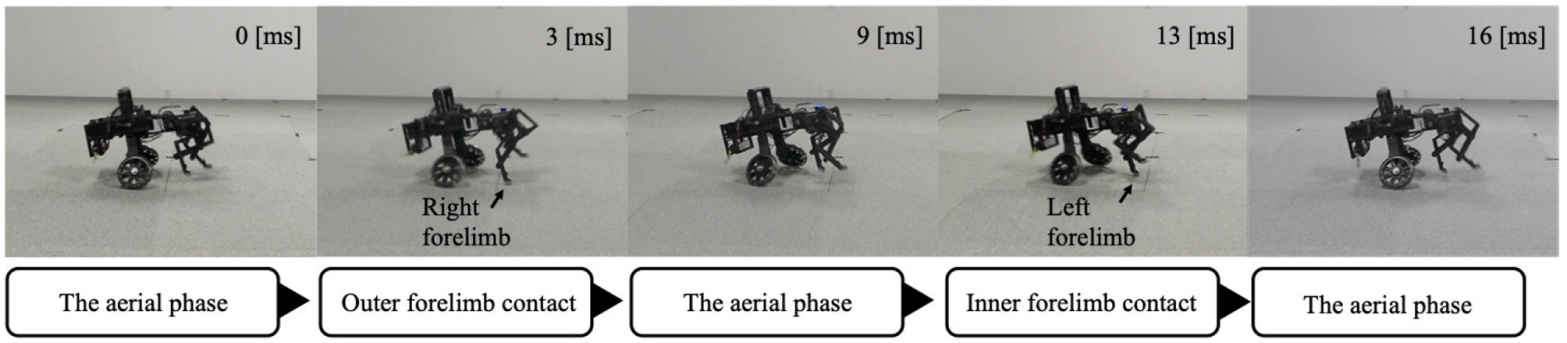
A snapshot of the robot from a lateral view during Condition 2. The outer and inner forelimbs alternately contact the ground.

The forelimb phase relationship illustrated in Figure 9C was approximately 1.0π [rad] before the yaw angle change and remained around 1.0π [rad] after the change. The footfall sequence depicted in Figure 9C indicates that, despite the yaw angle change, the outer leg (red) and the inner leg (blue) continued to contact the ground alternately, maintaining a symmetric gait (Supplementary Video S3). Figure 10 shows a side-view snapshot of the robot. After the swing phase of both legs, the outer left forelimb and the inner right forelimb made ground contact alternately. Notably, when only the yaw angle of the trunk was bent without changing the roll angle, the left forelimb on the inner side did not become the leading limb.

## 4 Discussion and conclusion

The significance of this study lies in demonstrating that physical interactions through the body also play a crucial role in galloping turns observed by quadrupeds. We confirmed that a trunk roll tilt is indispensable for turning, as the robot consistently turned toward the side of the tilt, with its inner forelimb spontaneously becoming the leading limb. In contrast, changes in trunk yaw angle alone did not induce such a emergence of leading limb. This result may be explained by the fact that trunk roll motion generates an asymmetry in limb-ground clearance, causing the inner limb to contact the ground relatively earlier. Therefore, the determination of the leading limb during turning is likely governed primarily by the physical interactions between the limbs and the ground induced by roll angle modulation. This determination of the leading limb by trunk roll inclination is consistent with previous studies showing that physical interactions through the body contribute to interlimb coordination during straight locomotion. The previously proposed interlimb coordination mechanism (Owaki et al., 2013) used for decentralized autonomous control of the forelimbs in this study has demonstrated that physical interactions among the body and ground is essential to reproduce gait transitions in straight locomotion. Considering the shared interlimb coordination mechanism in this and previous studies, our findings suggest that physical interactions are crucial for generating the adaptive coordination patterns observed in animals, both during straight locomotion and turning.

The robot experiments further indicate that trunk roll angle has a stronger influence on interlimb coordination than yaw-axis bending during galloping turns in quadrupeds; this aligns with previous kinematic and anatomical studies of animals. Kinematic studies on horses moving along small circular paths at a walk, trot, or canter have reported minimal variation in lateral bending angles of the cervicothoracic and thoracolumbar regions, while trunk inclination increases with gait speed (Egenvall et al., 2023). Anatomical studies indicate that spinal lateral bending rarely occurs in isolation and is often accompanied by axial rotation. For instance, a study on cats showed that axial rotational stiffness is lower than lateral bending stiffness, suggesting that much of the flexibility in lateral movements depends on rotational motions involving axial rotation (Macpherson and Ye, 1998). Similarly, axial rotation of the thoracic vertebrae has been observed during lateral bending in horses (Townsend et al., 1983). In this study, Townsend further suggested that utilizing spinal axial rotation, rather than independently controlling limb abduction and adduction, may enable more efficient and coordinated lateral swinging of the limbs. Collectively, these findings suggest that during high-speed galloping turns, trunk roll angle likely plays a more dominant role than yaw-axis bending in shaping interlimb coordination.

To better understand the turning mechanism, we need to investigate whether the control mechanism of this study can also be applied to a flexible trunk, since actual quadrupeds have multiple joint degrees of freedom (Denoix, 1999; Townsend et al., 1983; Macpherson and Ye, 1998). In our experiments, the hardware was specifically designed so that trunk yaw and roll could be independently adjusted, allowing us to examine which factor contributes more strongly to the emergence of the inside-leading limb. As a result, tilting the trunk roll angle alone led to the inner forelimb becoming the leading limb. We consider that this result may be explained by trunk roll tilting motion, in contrast to trunk yaw bending motion alone, creating an asymmetry in limb-ground clearance, resulting in the inner limb contacting the ground earlier. If such asymmetries in clearance between the limbs and the ground are maintained in a multi-joint trunk, the mechanism demonstrated in this study could also be applied in actual quadrupeds.

The wheeled hindlimb structure adopted in this study is considered to capture the essential features of forelimb coordination during galloping turns in quadrupeds. Even in cases where quadrupeds use wheelchairs to support their body in place of their immobile hindlimbs due to fractures or paralysis (Chansangsri and Thawesaengskulthai, 2014), asymmetric turning gaits driven solely by the forelimbs have been observed (Walkin' Pets, 8 30). In such cases, the inner forelimb tends to act as the leading limb and the outer forelimb as the trailing limb, similar to healthy quadrupeds (Jaxom Wolf, 8 30). These findings suggest that a robot with a wheeled hind module is adequate for examining forelimb coordination during galloping turns. As the next step, a mechanical update that includes active hindlimb structures is required to explore balance control with the body having a high center of mass and the role of the hindlimbs in different styles of gallop. For instance, while the forelimb footfall patterns are shared between transverse and rotary gallops, the hindlimb patterns differ: in rotary gallop, the inner hindlimb contacts the ground before the outer hindlimb, whereas in transverse gallop, the order is reversed. Future robotic platforms should allow controllable hindlimb contact and lift-off to investigate how these differences affect maneuverability and motor control across species.

Although our findings are based on thorough experiments using a small dog-sized robot, we anticipate that they can be generalized to medium- to large-sized quadrupeds adept at galloping. In our robot experiments, trunk roll modulation produced trunk leaning, resulting in the inner forelimb becoming the leading limb. In real-world animals of various sizes–including dogs, cheetahs, and horses–this strategy of leaning the trunk during turning (Higurashi et al., 2024; Ichikawa et al., 2018; Egenvall et al., 2023) and using the inner forelimb as leading limb (Higurashi et al., 2024; Ichikawa et al., 2018; Parkin et al., 2006; Williams and Norris, 2007) is consistently employed. We capture these similarities of both robot experiments and real quadrupeds, which suggests that leaning-in is key to the emergence of the inner-leading limb. Therefore, we expect that the proposed mechanism can be applied to quadrupeds of various scales, provided they are capable of leaning their bodies during turning (Biewener and Patek, 2018).

To better understand the agile turning of quadrupeds, in future work we need to investigate control mechanisms that allow trunk angles, such as roll and yaw, to flexibly vary as observed in animals. In this study, to simplify the experimental conditions, we controlled the trunk roll and yaw angles of the robot by fixing their target values. However, actual quadrupeds are known to exhibit dynamic oscillations and twisting of the trunk throughout the turning cycle (Byström et al., 2021; Egenvall et al., 2023). Such flexible behavior may be reproduced by incorporating sensory feedback. For example, in our previous study, we realized cheetah-like running that utilized trunk flexion-extension movements through bidirectional sensory feedback control between the limbs and trunk (Fukuhara et al., 2020a). We believe that this approach could also be applied to the control of trunk roll angles, which may enable more agile turning performance.

In this study, the targeted motion was limited to unidirectional turning during a gallop gait. Although the gait eventually converged such that the inner forelimb on the rolled side became the leading limb and the outer forelimb became the trailing limb, this convergence required four strides (Figures 6C, D). However, actual animals moving in a gallop gait along paths that combine different turning directions, such as a figure-eight (Higurashi et al., 2024), exhibit a phenomenon where they switch the leading limb in mid-air within one or two strides depending on the turning direction (Back and Clayton, 2013). Two key factors are essential to achieve instantaneous limb switching during the swing phase, as seen in actual animals: first factor is the ability to acquire and adjust postural information in response to the turning direction, which requires integrating sensory information from higher centers, such as the visual and vestibular systems; second factor is the ascending modulation that transmits information from the legs to the central nervous system, which is considered necessary to accurately grasp the state of the legs, such as which left or right leg is currently landing first, for switching the leading limb in mid-air. As future work, understanding directional changes in biological gallop requires incorporating elements of higher centers and addressing control mechanisms in which ascending and descending modulations interact.

## Data Availability

The raw data supporting the conclusions of this article will be made available by the authors, without undue reservation.
