# Supplementary material for: A simple robot suggests trunk rotation is essential for emergence of inside leading limb during quadruped galloping turns
Source: Front Neurorobot. 2025 Oct 23;19:1628368. doi: 10.3389/fnbot.2025.1628368 (PMC12590251; doi:10.3389/fnbot.2025.1628368)
Supplement: Supplementary file 1 [file Data_Sheet_1.pdf]

Supplementary Material

1 ACQUISITION OF GROUND REACTION FORCE INFORMATION

In this study, the servo motor (Dynamixel XM430-W350-R) used as the leg actuator provides a current value proportional to motor torque. Previous studies have used the motor's current value at the elbow (knee) joint (with extension as positive) to estimate leg load from ground reaction force(Sun et al., 2020; Amaike et al., 2023). We aimed to determine the magnitude of body weight support during the leg's stance phase using the servo motor current. However, we implemented a dead zone for accurate leg load acquisition since current also arises from leg movements during the swing phase of locomotion. The dead zone was established through the following procedure:

1. With the body off the ground, we moved the legs using anti-phase feedforward control to obtain the current value during the swing phase.
2. We ran the robot on a treadmill using anti-phase feedforward control to obtain current values for both the swing and stance phases.
3. We established the threshold for the dead zone using the current values obtained during movements in step 1 (with the body off the ground) and step 2 (during treadmill locomotion) and determined the lower and upper bounds of the threshold, I_{bottom} and I_{upper}, respectively. The result from step 1 and step 2, the shoulder servomotor's current value changed significantly upon ground contact; we used this motor's current value to represent ground reaction force (Fig.S1). Specifically, the sensor value S_i is defined as follows:

$$S_i = \begin{cases} 0 & (\text{if } I_{bottom} < I_i < I_{upper}) \\ I_i & \text{otherwise} \end{cases}$$

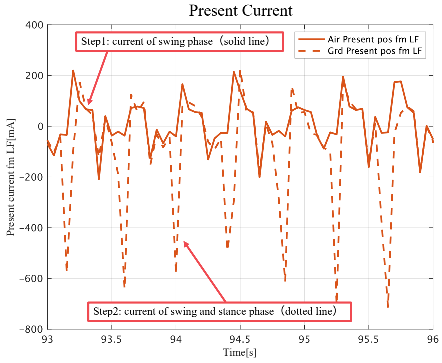

Figure S1. Method for isolating ground reaction force from motor current measurements. The graph shows the motor current during the swing phase (Step 1, solid line) and during both swing and stance phases (Step 2, dotted line), used to differentiate the current component related to the ground reaction force.

Since the current values in step 1 stabilized within a specific range, we set $I_{bottom} = -225$ [mA] and $I_{bottom} = 270$ [mA], respectively. Each limb was controlled by two motors whose roles in fore-aft

movement and flexion-extension are not completely separated; the two motors cooperate to generate the leg trajectory through inverse kinematics.

2 CALCULATION OF THE TURNING RADIUS

The robot approximately followed a circular trajectory, and the y-coordinate was partially out of frame for some parameter settings. Therefore, the turning radius R was subsequently calculated by categorizing the data based on the maximum ($M = \max(x)$) and minimum ($m = \min(x)$)x-coordinate values within the time period T (Fig.S2):

$$R = \begin{cases} \frac{|M|+|m|}{2}, & \text{if } M > 0 \text{ and } m < 0 \\ \frac{|M|-|m|}{2}, & \text{if } M > 0 \text{ and } m > 0 \\ \frac{|m|-|M|}{2}, & \text{if } M < 0 \text{ and } m < 0 \end{cases}$$

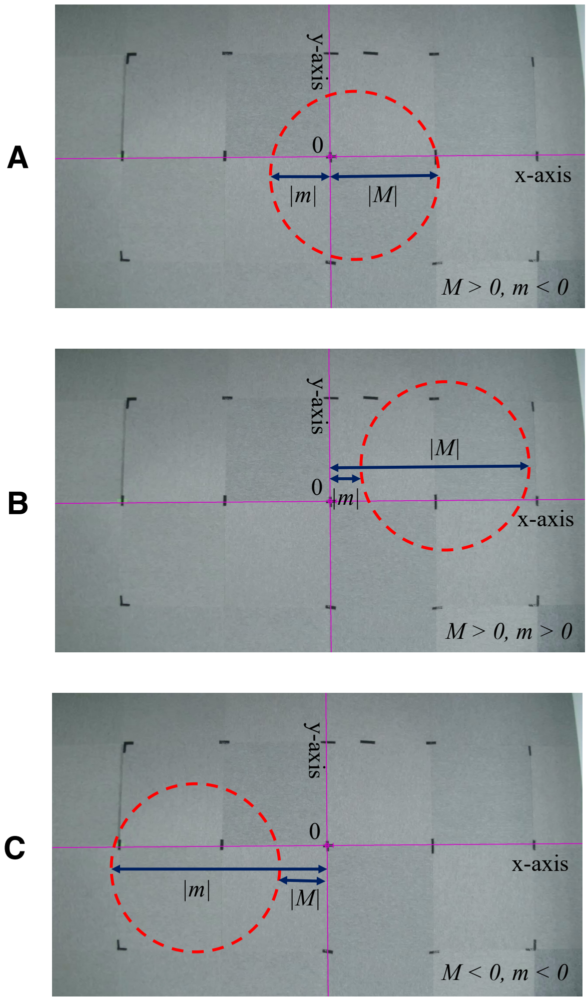

Figure S2. Calculation of the turning radius. **(A)** In the case where $M > 0$ and $m < 0$. **(B)** In the case where $M > 0$ and $m > 0$. **(C)** In the case where if $M < 0$ and $m < 0$.

REFERENCES

Amaike, H., Fukuhara, A., Kano, T., and Ishiguro, A. (2023). Decentralized control mechanism underlying morphology-dependent quadruped turning. *Journal of Robotics and Mechatronics* 35, 1290–1299

Sun, T., Xiong, X., Dai, Z., and Manoonpong, P. (2020). Small-sized reconfigurable quadruped robot with multiple sensory feedback for studying adaptive and versatile behaviors. *Frontiers in neurorobotics* 14, 14
